# A Wide Dynamic Range Polarization Sensing Long Wave Infrared Detector

**DOI:** 10.1038/s41598-017-17675-6

**Published:** 2017-12-12

**Authors:** Elham Mohammadi, Nader Behdad

**Affiliations:** University of Wisconsin-Madison, Department of Electrical and Computer Engineering, Madison, WI 53706 USA

## Abstract

We present the design, fabrication, and characterization of an infrared (IR) polarization sensing detector with a wide dynamic range and sub-wavelength dimensions. The detector consists of two orthogonal slot antennas, each loaded with two microbolometers at its edges. The polarization of the incoming IR radiation is detected by comparing the received power levels in the bolometer pairs corresponding to each slot antenna. The IR radiation is sensed by applying a dc bias voltage to each antenna and measuring the changes in the dc current caused by the change of the bolometer resistance as they absorb the incoming IR radiation. In this design, the ratio of the absorbed power in the bolometers is a one to one function of the polarization of the incident wave. A prototype of this detector, designed to have maximum sensitivity at *λ* = 10.6 *μ*m, was designed, fabricated, and characterized. The fabricated detector has an area of 0.7*λ* × 0.7*λ*, where *λ* is the free-space wavelength. The polarization sensing response is characterized under different angles of incidence. The measurement results show that the device has a dynamic range of 24 dB between two orthogonal orientations of EM wave polarization for incidence angles in the range of ±20° from boresight.

## Introduction

The spectrum of thermal radiation emitted from an object is dependent on its temperature. The majority of the thermal energy radiated from objects that are at room temperature (300 K) is in the long wave infrared (LWIR) window with maximum radiation power occurring at around *λ* = 10.6 *μ*m. Imaging systems working at LWIR frequency bands can use this thermal radiation for sensing without using any extrinsic sources. These IR imaging systems have a variety of applications in military such as night vision monitoring and target tracking, and in science and industry such as non-contact temperature measurement^1–6^. current IR detector technology is divided into two main categories of photon and thermal detectors. Photon detectors such as HgCdTe and quantum-well photodetectors have superior performance in terms of better detectivity and faster response times compared to thermal detectors but they must be cooled to very low temperature levels (often cryogenically). Thermal detectors, such as thermopiles and bolometers have the advantage of being lightweight and reliable sensors capable of working at room temperature^7^. Regardless of the method of sensing (thermal or photon detection), most available detectors primarily sense the irradiance of the incoming wave and ignore other attributes of the wave such as polarization or angle of arrival. Sensing these additional attributes of the incoming wavefront may offer additional information that can be exploited to perform sensing and imaging in visually degraded environments^8,9^.

Polarization sensing systems are used for many applications such as facial recognition^10^, target tracking^11^, remote sensing^8^, and astronomy^12^. The techniques used in these systems require use of either auxiliary elements, such as quarter wave plates^13^ and polarizer filters^14^, or using integrated micropolarizers at the pixel level^15–17^. The use of external components is not the ideal approach as they are bulky and attenuate the incident wave. For example, using polarizing filters results in wasting half of the energy of the incoming wave since photons whose polarizations are not matched to that of the polarizing filter are either attenuated or reflected. Additionally, in such systems the performance of the detector is sensitive to the alignment between the detector and these components. Nevertheless, for IR imaging systems, these external components are currently preferred over the use of integrated micropolarizers, because the dimensions of these integrated micro-elements are usually larger than *λ* ^2^, which results low resolution images^18^. The use of micropolarizers is more popular at higher frequencies (e.g. in the visible range), where the wavelength is much smaller than that at the LWIR domain^15,16^.

A third and less-explored approach for designing polarization sensing IR detectors is to use antennas that are polarization selective and are coupled to thermal detectors such as bolometeres^19^, MIM diodes^20^, and thermopiles^21^. In this method, the IR currents induced on the antenna by the incoming IR wave are absorbed in the thermal detector. Since the strength of the currents induced on the antenna depends on the polarization of the incoming wave, the power absorbed in the thermal detector will also be polarization dependent. By using appropriate, dual-polarized antenna designs, one can realize detectors capable of sensing both the intensity and the polarization of an incoming IR wave using a detector with sub-wavelength physical dimensions. Since the effective area of a sub-wavelength antenna is usually substantially larger than its physical occupied area^22^, these detectors will have collection (or capture) areas considerably larger than the physical areas they occupy. Antenna-based, polarization sensing is usually accomplished using two antennas with orthogonal polarizations. By comparing the absorbed power in each antenna, the polarization of the incoming EM wave can be detected regardless of the irradiation level as long as the level of the received signal is sufficiently above the noise level. Krenz *et al*. used two orthogonal dipole antennas coupled to microbolometers to perform polarization sensing^23^. The absorbed power in each dipole antenna is a function of power and polarization of incident beam. However, since they measured the power difference between the two antennas, the total polarization response is still proportional to the incident power. Furthermore, bonding pads and other external measurement devices in their design may also change the response of each individual dipole. Another study used various combinations of dipole and spiral antennas^24^ to measure the Stoke parameters. However, the reported results for each antenna is only valid for a constant incident power and would change under a different of the incident power. In this study, the output signal from the measurement of each configuration is the sum of the response from the two orthogonal antennas. In another study, a response matrix analysis was used to measure the polarization response, independent of the incident power^25,26^. They have used two orthogonal bowtie photoconductive antennas for THz time-domain spectroscopy. However, this type of detectors are suitable for terahertz pulses detection, and are not applicable for infrared detection. Also, these structures employ long aspect ratio lead lines, which degrade the polarization response of the system^26^. Therefore, even though the authors have considered the effect of bias line in their matrix analysis, any change in the bias line configuration such as length, direction, or thickness, degrades the device’s response.

**Figure 1:**
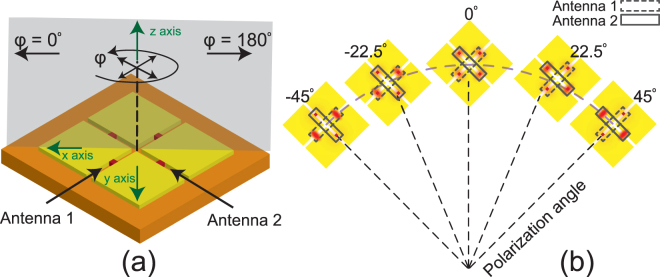
(**a**) Schematics of the polarization sensing detector using slot antennas coupled to bolometer loads. Antenna 1 receives the maximum power when the incident beam has an electric field in the plane of *ϕ* = 135° and *ϕ* = 315° and Antenna 2 has the maximum absorption when the electric field is in the plane of *ϕ* = 45° and *ϕ* = 225°. (**b**) The behavior of bolometer loads in the orthogonal antennas during the polarization sensing process. The red glows around the bolometers represent the temperature of each bolometers. Higher intensity glow indicates higher temperatures. At polarization angle of zero, bolometers of both antennas receive equal amounts of power. The temperature of bolometers in slot antenna 1 (2) are higher when the polarization angle is −45° (45°).

In this work, we present a new design for a polarization sensing infrared antenna, which offers a wide dynamic range and is less sensitive to the adverse impacts of bias lines. The topology of the proposed device, which consists of two orthogonal rectangular slot antennas, is shown in Fig. 1(a). Slot antennas coupled to IR detectors, such as bolometers^27,28^ and MIM diodes^29^ have been examined in the past. Slot antennas offer desirable traits such as fabrication ease, higher gains, and wider bandwidths^28^. However, most of these previously presented designs^27,28^ do not sense the polarization of the incident wave and were proposed as simple antenna-coupled IR detectors. In another work, two arrays of orthogonal slot antennas coupled to microbolometers were used to design a dual polarized detector for submilimeter imaging^30^. Their detector was designed for lower frequencies than IR, which resulted in larger feature sizes and easier fabrication procedure. This allowed them to use a separate layer for wiring their bolometers and to use a simpler design. However, this design is not applicable to IR region due to fabrication limitations. Also, the low resistance of bolometers was not properly matched with the relatively high input resistance in the middle of the slot antennas, which prohibits maximum power absorption.

Here, we present a polarization sensitive IR detector capable of detecting the polarization angle regardless of the irradiance. Two sets of microbolometers serve as both the thermal detectors^31^ of the IR radiation and as physical parts of the antenna defining the slot length and hence, its resonant frequency. This novel use of bolometers as structural parts of the slot antennas themselves significantly simplifies the IR/dc separation requirements. Since slot antennas absorb the EM radiation best if the electric field of the incident wave is perpendicular to the slot, they provide excellent polarization sensitivity^32^. A dc voltage is applied across the microbolometers and the dc current passing through each bolometer is measured. The IR currents induced on the ground plane of the slot antennas are absorbed in the microbolometers causing their temperatures, and hence their resistances, to change. Therefore, monitoring the changes in the dc current passing through each pair of bolometers determines the level of the power absorption in the corresponding slot antenna. As a result of the structure of the device and the use of slot antennas connected to large ground planes, we do not require any bias lines and the device is directly connected to large biasing pads (not shown in Fig. 1). This has minimal effect on the polarization selectivity of the device and results in polarization selective response much better than those of the previously reported devices^23–25^. For each polarization angle, the bolometers respective to each slot antennas show different temperature variations. As shown in Fig. 1(b), when the infrared beam with polarization angle of *ϕ* = 45° is incident on the device, antenna 2 with slot direction perpendicular to the electric field receives maximum power. In contrast, antenna 1 with slot direction parallel to the electric field absorbs comparatively small radiation power. Therefore, the temperature variation of bolometers in antenna 2 are much higher than those of antenna 1. As the polarization angle decreases, the temperature variation of bolometers in antenna 2 decreases and that of antenna 1 increases and the temperature variations become identical for *ϕ* = 0°. The same process repeats for −45° < *ϕ* < 0° however this time the temperature variation for bolometers in antenna 1 is higher. Therefore, by comparing the relative changes of resistances of the bolometers, one can determine the polarization of the incident EM wave. By using the slot antennas and the ratio of the variations in temperature, we have managed to drastically reduce the effects of the external measurement setups, bias lines, and the irradiance of the EM wave on the final response function of the detector. The total total physical area occupied by the two antennas in the proposed design is 0.7*λ* × 0.7*λ*, where *λ* = 10.6 *μm* is the free-space wavelength at the center frequency of operation.

## Results

### Design and optimization

**Figure 2:**
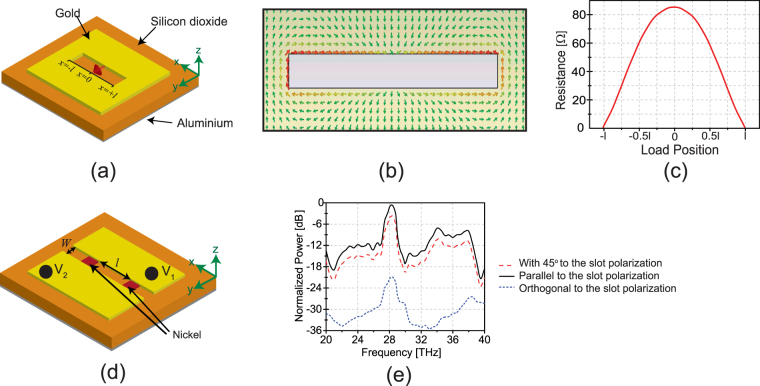
Design procedure for a single slot antenna coupled to microbolometers. (**a**) Schematic of a conventional slot antenna with a port in the middle, which represents the load. (**b**) Electric current distribution around the slot. Current is maximum at the edges of the slot and zero at the center of the slot. (**c**) The input resistance of the slot antenna changes with moving the position of the port along the slot opening. By moving towards the edges of the slot, the input resistance decreases. (**d**) Schematic of the modified structure of the slot antenna coupled with two microbolometers used as the detectors of the IR wave. The areas beyond the bolometer loads are removed to provide the dc isolation between the two sides of each bolometer. The path for induced IR current in the antenna is provided by metallic bolometers. (**e**) The absorbed power in the bolometers as a function of incoming wave frequency for different states of polarization. The distance between the two bolometers, which determines the slot length is optimized to receive the maximum power at the working frequency of 28.3 THz.

In our design of the polarization sensing infrared detector, two orthogonal slot antennas are used. Each slot antenna is designed separately and then both slots are combined together and their performance was determined and optimized by computer simulations. In this section, we explain the design procedure. The first step is to design a conventional slot antenna (shown in Fig. 2(a)), which resonates at the desired IR frequency of 28.3 THz corresponding to a free-space wavelength of *λ* = 10.6 *μm*. Each slot antenna is defined in a gold ground plane placed on top of a 700 nm-thick silicon dioxide layer. This value was chosen to optimize the performance of the antenna and to improve the fabrication quality and yield of the device. Using thicknesses below 700 nm causes wire bonding ploblems and degrades the system performance (See the Supplementary materials Fig. S1 to see the effect of the dielectric thickness on the pefromance of the device). Also, as we increase the thickness of the layers during the fabrication, the roughness of the deposited layer increases, which results in lower yield in fabrication. The bottom side of the *SiO*_2_, layer is completely covered with a 200 nm-thick layer of aluminium, which serves as a floating ground plane for the slot antenna. This layer is different from the ground plane sourounding the slots, which is made of gold. The slot length is determined using the design equation 1, where *L*_*slot*_ is the slot length, *λ*_0_ is the free-space wavelength and *ε*_*eff*_ is effective relative permitivity of the structure, which has a value between the relative permitivity of *SiO*_2_ and one. In determining the length of the slot, the material parameters for gold and *SiO*_2_ obtained from the published literature^33,34^, were used. The physical length of the slot was determined to be 4.4 *μ*m to achieve resonance at 28.3 THz.

1 $${L}_{slot}=\frac{{\lambda }_{0}}{2\sqrt{{\varepsilon }_{eff}}}$$

The next step is to find the optimum position for the bolometer loads, which maximizes the absorbed IR energy in the bolometers. Figure 2(b) shows electric current distribution in the ground plane surrounding the slot antenna shown in Fig. 2(a). Observe that the intensity of the electric current is strongest at the edges of the slot. Intuitively, this can be explained by modeling a slot antenna as a transmission line (slot line) short circuited at its both ends. In such a structure, the maximum value of the standing-wave electric current occurs at the short circuit locations (edges of the slot antenna or the short circuited points). Therefore, placing bolometers at these locations maximizes the amount of energy absorbed and results in a more pronounced change in temperature for a given bolometer and a given incident power density of the IR wave. This choice of bolometer location can also be justified by examining impedance matching considerations. Specifically, the resistive bolometers used in this design have low impedance values (in the range of few Ohms). Therefore, placing them along the edges of the slot antenna allows for achieving a better impedance match and maximum power transfer between the antenna and the load.

As we can see in Fig. 2(a), a conventional slot antenna has a continuous metallic ground. When a bolometer is used as a load for this slot antenna, both of its terminals are dc connected to each other because the slot antenna of Fig. 2(a) is etched in a continuous ground plane. This presents a practical problem since the resistance change of the bolometers are detected by applying a constant dc voltage across the bolometers and measuring the variation of the dc current passing through them. Therefore, a suitable IR/dc isolation mechanism needs to be utilized that allows for a continuous flow of the IR currents in the ground plane of the slot antenna around the slot, yet provides a distinct, controlled path for the dc current to flow through the bolometers. To accomplish this, we propose the new slot antenna topology shown in Fig. 2(d). In this structure, the two halves of the ground plane of the slot antenna are completely separated from each other by a distance of *w* (the width of the slot antenna). The slot antenna itself is defined by the two bolometers placed between these two half ground planes. The separation between the two bolometers defines the resonant length of the slot antenna. This structure supports the IR current flow in the ground plane of the slot. At its resonance, the IR current intensity is maximum at the location of the bolometers, which enhances the power absorption in the bolometers. At the same time, when the two half ground planes are connected to dc bias voltages of *V*_1_ and *V*_2_, a dc current must pass through the two bolometers. Since the dc resistance of the bolometers is higher than that of the wide ground planes, the changes of the bolometer resistance can be monitored conveniently using this topology. Figure 2(e) shows the normalized power absorption in the bolometers for the slot antenna shown in Fig. 2(d) as a function of frequency. Observed that the absorbed power is maximized at 28.3 THz (10.6 *μ*m) indicating that the slot antenna is strongly resonant at this frequency. To the best of our knowledge, this is the first time that this antenna-coupled-bolometer topology is reported.

The antenna shown in Fig. 2(d) absorbs the maximum power level at its resonance when the polarization of the incoming EM wave is orthogonal to the slot (i.e., the electric field is oriented along the $\hat{y}$ direction). When the polarization of the incident field is parallel to the slot, the absorbed power level decreases significantly. Specifically, as shown in Fig. 2(e), the absorbed power level decreases by two orders of magnitude for the orthogonal polarization. Reducing the width of the slot antenna (*w*) further increases this polarization purity. However, reducing the slot width reduces the bolometer resistance for a fixed bolometer width. This results in reduction of the variations of the resistance caused by the absorption of the incoming IR radiation which reduces the magnitude of the desired output signal and hence, the detectivity of the device. Moreover, decreasing *w* also reduces the bandwidth of the antenna^35^. Therefore, there is a tradeoff between the bandwidth, polarization purity, and detectivity of the device. Here, we used a 600 nm wide slot, which leads to the impedance of ~15 Ω for each microbolometer (with thickness of 100 nm and width of 600 nm) and 19 dB power difference between the two orthogonal states of polarization.

**Figure 3:**
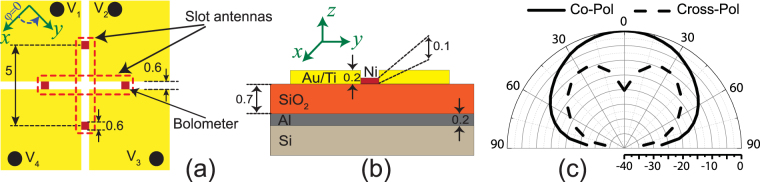
The structure and dimensions of the polarization sensing detector. (**a**) The top view of the two orthogonal slot antennas each coupled with two microbolometers. The two antennas provide polarization sensing and the two bolometers are placed to maintain the symmetry of the structure and provide the path for dc and IR currents. The dimensions of different elements are also shown. (**b**) The cross section view of the detector. The device is designed on a silicon dioxide dielectric and an aluminum ground plane. All dimensions are in micrometers. (**c**) Simulated radiation patterns of the antenna for the co-pol and cross-pol components. The absorbed power in the co-polarized state is reduced as the angle of incidence increases. On the other hand, the absorbed power of cross-polarized state increases as the angle of incidence increases and reaches its peak at *θ* = 30°.

While this slot antenna has a good polarization purity, it cannot be used alone to determine the state of the polarization of the incoming wave, since the change in the absorbed power can be due to the change of polarization or the irradiance of the incoming wave. To detect the polarization of the incident EM wave irresprective of its irradiance, another identical slot antenna is placed orthogonal to the first slot as shown in Fig. 3(a). One antenna responds strongly to incoming waves whose electric fields are parallel to the plane of *ϕ* = 45° and the other one has its peak sensitivity for waves polarized along the *ϕ* = −45° direction [Fig. 1(a)]. Therefore, by monitoring the ratio of the absorbed powers in the bolometers corresponding to the two slot antennas, we can detect the polarization of the incoming wave without any information about the irradiance of the wave. Figure 3(a) shows the topology of the final design of the detector and the dimensions of all components. The cross sectional view of the detector with the thicknesses of the different layers of materials are shown in Fig. 3(b). In order to predict the response of the detector for different incidence angles, the radiation patterns of the one of the slot antennas shown in Fig. 3(a) is presented in Fig. 3(c). This slot antenna has a 13 *μm* × 13 *μm* finite ground plane and the ground plane of the slot is truncated from the middle to define the other slot antenna. In order to extract the co-pol (cross-pol) components of the radiation pattern, the antenna was excited with a plane wave with electric field parallel (orthogonal) to the slot antenna’s polarization. The angle of incidence of this plane wave was changed and the absorbed power in the bolometers of the antenna were monitored. In these simulations, the angle of incidence is in plane between the *z*-axis and the primary axis of the slot. As can be seen, as the angle of incidence increases, the co-pol component reduces and the cross-pol component increases. Therefore, the power difference between the two orthogonal states of the polarization, i.e. the dynamic range, reduces. The co-pol and cross-pol patterns shown in Fig. 3(c) are similar to those of a conventional slot antenna with an infinite ground plane size. Low-frequency slot antennas with finite ground plane dimensions tend to have oscillatory radiation patterns caused by the out-of-phase radiation of the electric currents from the ground plane edges^36^. This phemomenon, however, is not observed in this design because the lossy nature of gold at IR frequencies significantly attenuates the strength of such currents, thereby minimizing their impact on the radiation patterns of the antenna.

### Simulation and Measurement Results

**Figure 4:**
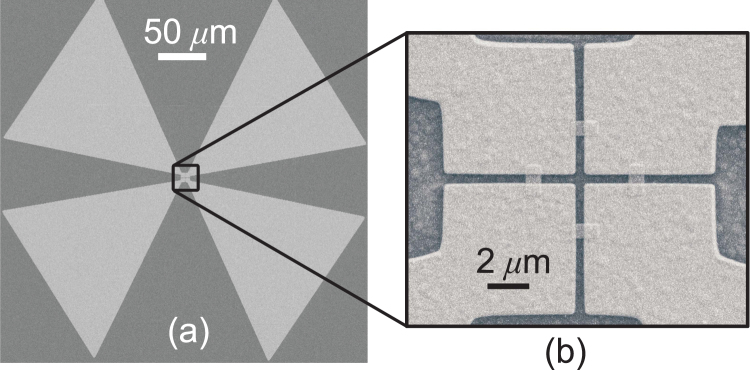
The scanning electron microscope (SEM) image of the fabricated prototype. (**a**) The detector is at the center of the figure which is connected to four large triangular pads that are used for the dc biasing of the bolometers. (**b**) The inset shows the magnified image of the slot antennas with four microbolometers.

We simulated the prototype device for different polarization angles and various angles of incidence for the incoming wave. The device was fabricated using standard clean-room microfabrication techniques. Figure 4 shows the scanning electron microscope (SEM) image of the fabricated device. In Fig. 4(a), the large triangular areas are the bias pads, which are directly connected to the ground planes of the slot antennas [see Fig. 3(a)]. The slot antennas and the bolometers are located at the center of the image [Fig. 4(b)]. Details of the computer simulations, fabrication process, and measurement setup are discussed in Methods section. During the measurement process, constant dc bias voltages were applied between the different pairs of ground planes, [*V*_1_–*V*_2_ or *V*_3_–*V*_4_ and *V*_1_–*V*_4_ or *V*_2_–*V*_3_ in Fig. 3(a)] to measure the response of each antenna separately, through measuring the resistance change. However, each measured value is a result of the resistance variations in the bolometers corresponding to both the horizontal and the vertical antennas. For example, in order to measure the resistance change in the bolometers in Fig. 3(a), we measure the resistance changes seen between ground planes *V*_1_–*V*_2_ (Δ*R*_12_) and *V*_2_–*V*_3_ (Δ*R*_23_). By assuming equal resistance change in the bolometers corresponding to each antenna, we can show that

2 $${\rm{\Delta }}{R}_{12}=\delta {r}_{{\rm{v}}}+\frac{\delta {r}_{{\rm{h}}}}{5},\quad {\rm{\Delta }}{R}_{23}=\delta {r}_{{\rm{h}}}+\frac{\delta {r}_{{\rm{v}}}}{5},$$

where *δr*_v_ (*δr*_h_) is the resistance change in bolometers of the vertical (horizontal) antenna. Therefore, we can easily calculate *δr*_v(h)_ by simple algebraic operations. For a detailed discussion of our method used to extract the resistance change in each individual bolometer refer to the Supplementary materials.

**Figure 5:**
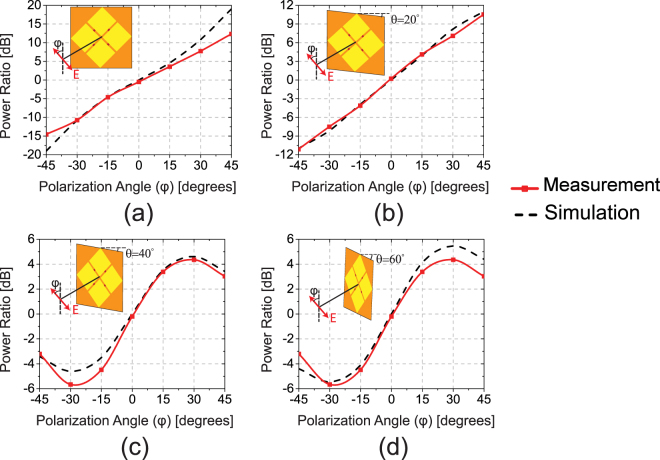
Simulation and measurement results of the polarization sensing IR detector for various incidence angles of (**a**) *θ* = 0°, (**b**) *θ* = 20°, (**c**) *θ* = 40°, (**d**) *θ* = 60°.

Figure 5(a–d) show the simulated and measured power ratios of the two antennas as a function of the polarization of the incident wave. The results are shown for different incidence angles, *θ*, measured with respect to the direction normal to the antenna plane (i.e., the *z* axis). For normal incidence, *θ* = 0°, the power ratio of the device is a one-to-one function of the incidence angle and it has a simulated dynamic range of 38 dB. The measured response is also in relatively good agreement with the simulation results. For the incidence angle of *θ* = 20°, the device’s response is still a one-to-one function of the polarization of incidence. However, the dynamic range is reduced from 38 to 21 dB. The measured response is also in relatively good agreement with the simulation results. When the polarization of the incident wave is in parallel to the polarization of one of the slot antennas, i.e. *ϕ* = ±45°, one of the antennas senses the electric field as its co-pol and the other as its cross-pol. In the case of *θ* = 20°, the co-pol component is no longer in its peak level and also the cross-pol level is elevated compared to the case for the normal incidence (see Fig. 3(c)). Therefore, the dynamic range of the polarization detection response decreases (compare Fig. 5(a) and (b)). Figure 5(c) and (d) show the simulated and measured responses of the device for incidence angles of *θ* = 40° and 60°. As can be seen, as the incidence angle is increased, the device’s response is no longer a one-to-one function of the polarization and the dynamic range decreases further. This trend can also be seen by examining the co-pol and cross-pol radiation patterns of a single slot antenna shown in Fig. 3(c).

As can be observed in Fig. 5, the measurement and simulation results are in good agreement. The small differences between them are attributed primarily to the fabrication imperfections and various sources of noises, such as resistor Johnson noise of the device and flicker and shot noise of measurement equipments^37,38^. The misalignment of the slots with bolometers during the lithography results in an asymmetric device. Therefore, at polarization angle of zero where both antennas sense an even portion of the incoming wave, the bolometers of the asymmetric device absorb uneven power. Since, we only measured the absorbed power of one of the bolometers in each slot antenna, the measured power ratios at this polarization are not exactly 0 dB. On the other hand, various types of noises such as junction noise, shot noise, and flicker noise are present during the measurements. These unavoidable noise sources are in the range of output signal when the total absorbed power in bolometers is low such as higher angle of incidents and when the polarization of incoming wave is not parallel to the polarization of the slot antennas. As a result, the measurements at higher polarization angels (i.e. *ϕ* ≥ 40°) shows the highest disagreement with the simulations.

To compare the responsivity of the infrared detectors, various figures of merit such as signal to noise ratio (SNR), noise equivalent power (NEP), and normalized detectivity (D*) are defined. To calculate all of these figures of merit, first we need to measure the noise of the device and measurement equipment such as bias voltage source, current preamplifier, and lock-in amplifier. To this end, we measured the noise from trial resistors with different values. The output signals were the sum of the noise of measurement equipment as well as Johnson noise, shot noise and flicker noise of the resistors. Using these measured signals and the fact that the resistors’ noises are the function of their resistance values, we extracted the noise from the measurement equipment. More information about the noise measurements is provided in the supplementary materials section. Then, we measured the output signal from the device under laser illumination. Therefore, the noise of the device can be extracted using the measured value in the second step and the calculated noise from measurement equipment. As a result, the measured noise includes all noise sources such as Johnson, flicker and shot noises.

The calculated noise and measured output signal results in an SNR value of 390. This value was measured, when the laser output power was 340 mW and the calculated beam diameter was 1 cm, under normal incidence. SNR can be used to calculated the *NEP* = *ϕ*_*e*_/*SNR*, where *ϕ*_*e*_ is the power incident on the detector and is equal to 108 nW. Therefore, the calculated NEP is 0.27 nW. Another figure of merit, D*, is defined as: ${D}^{\ast }=\sqrt{{A}_{eff}\times BW}/NEP$, where *A*_*eff*_ is the effective area of the detector and BW is bandwidth of measurement equipments. Using this formula, the detectivity of the detectors is $5.8\times {10}^{5}\,cm\times \sqrt{Hz}\times {W}^{-1}$. This value is comparable to those of the other reported antenna coupled bolometers^39^.

## Discussion

A polarization sensing infrared detector consisting of two orthogonal slot antennas was presented and discussed in this paper. The detector can simultaneously sense the intensity and the state of polarization of an incomign IR waefront. Each slot antenna used in the proposed detector exploits a new antenna-coupled-bolometer architecture where two microbolometers are used to both sense the incoming IR radiation and define the resonant length of the slot antenna. This unique topology, which to the best of our knowledge is reported for the first time in our work, eliminates the need for using long bias lines that are commonly used in previously reported antenna-coupled-bolometers^22,40,41^. Elimination of long dc bias lines also improves the polarization purity of the device since these lines usually have polarization dependent responses themselves. A prototype of the device was fabricated using the standard microfabrication techniques in a clean room facility. The fabricated prototype has a measured dynamic range of more than 22 dB for incidence angles close to normal. The slot antenna prototype presented in this work has finite ground plane dimensions of 13 *μm* × 13 *μm*. Due to the lossy nature of metals at IR frequencies, the ground plane dimensions of the antenna can be further reduced following techniques reported in the literature^36^ without significantly impacting the performance of the proposed detector.

To simplify the fabrication, the fabricated device presented in this work uses nickel microbolometers and silicon dioxide as the antenna substrate. This was done here since our focus in this work was to present a proof-of-concept prototype and to introduce this new dual-polarized, IR antenna architecture. However, in the future, the performance of a device of this type can be enhanced by using bolometers with higher temperature coefficient of resistance, such as vanadium oxide^42^ and by thermally isolating the bolometers from the substrate of the slot antenna using micromachining techniques^39^. Additionally, lower loss materials may also be used as the antenna substrate instead of the silicon dioxide used here. Examples include benzocy-clobutene (BCB) polymer^41^ and zirconium dioxide (*ZrO*_2_)^43^. The paper also demonstrated that the performances of the proposed detector can be predicted to a very good degree of accuracy by using full-wave electromagnetic simulations as evidenced by the good agreements observed between the measurement and the simulation results.

## Methods

### Computer Simulations

The performance of the designed device was simulated and optimized using the commercial full-wave electromagnetic simulation software, CST Microwave Studio. In all simulations, the complex permittivity of different materials, obtained from various sources in the literature^33,34,44,45^, were used. All different parts of the detector with dimensions shown in Fig. 3 were included in the simulations. In order to simplify the simulations, a PEC boundary at the bottom (below the floating aluminum ground plane), open space boundary on the top, and open boundary on the other directions were defined around the device.

Time-domain simulations were used to find the input impedance shown in Fig. 2(c) and absorbed power of a single antenna at different frequencies as shown in Fig. 2(e). However, due to the faster response of single frequency simulations, the frequency-domain engine of CST was used in other simulations shown in Fig. 5. In all simulations, the incident beam was modeled with a plane wave. The direction of the plane wave determines the angle of incidence and its electric field direction dictates the angle of polarization. Using the illuminated plane waves, the electric field distribution all over the simulation volume including the volume within the bolometers and the different materials constituting the antenna was calculated by solving the Maxwell’s equations. Using the solutions of Maxwell’s equations obtained in this process, the total ohmic power loss in each of the bolometers was then calculated by integrating the power loss density throughout the volume of each bolometer (CST automatically performs this process). The absorbed power in bolometers were monitored and the ratios of the power absorbed in bolometers of the two slot antennas was calculated for different angles and polarizations.

### Fabrication

The whole fabrication process of the polarization detector was performed on a high resistivity silicon wafer. First, the wafer was coated with 200 nm aluminum for ground plane, using dc plasma sputtering. Then, a 700 nm-thick silicon dioxide was deposited by electron-beam evaporator. For patterning antennas and bolometers, electron-beam lithography and conventional liftoff methods were performed. For better quality of lithography, such as more reliable and easier lift-off, two layers of positive resist consist of methyl methacryllate (MMA) copolymer followed by polymethyl methacrylate (PMMA) were spin coated. 150 nm-thick gold antennas and 100 nm-thick Nickel bolometrs were deposited by electorn beam evaporator. Furthermore, a titanium layer with thickness of 50 nm was deposited beneath the gold layer, which increases the pure adhesion of gold to silicon dioxide. At the end, for measurement purposes, the device was wire bonded to a chip holder.

### Measurement Setup

The fabricated device was placed on a two dimensional motorized translation stage (two in-house assembled Thorlab MT1-Z8), which was connected to a rotational stage (Thorlab PR01). The device was illuminated by a CO_2_ laser tuned at 10.6 *μm* with up to 1 W. A wire grid polarizer (Thorlab WP25M-IRC) was placed in front of the laser to change the randomly polarized laser beam to a linearly polarized one. This wire grid polarizer was mounted on a rotational stage (Thorlab RSP1), which was used to change the polarization angle for different states of measurements. The polarized beam was passed through a Thorlabs ZnSe plate beamsplitter. Part of the beam was directed to a power meter and the other part was directed to an SR540 Stanford Research System chopper, which mechanically chopped the beam at 400 Hz. Then, the beam was illuminated to the detector. The detector under test was externally connected to a bias voltage in series to a current preamplifier (model SR570 from Stanford Research System). The voltage source provided a constant voltage at each bolometers and the current preamplifier was used to amplify the current passing through the bolometers and also to filter out the lower frequency components of current. The output of the preamplifier was connected to a 7230 Signal Recovery lock-in-amplifier, which was referenced to the chopper frequency. The lock-in-amplifier, demodulate the current variation from a noisy output signal.

In all measurements, the device was aligned using an automated computer controlled process. A computer controls the movement of the motorized stage and records the output signal of a single bolometer. The device is then moved to a new position and the process is repeated for 36 points in a 0.5 mm × 0.5 mm rectangular grid. Through this process, the incident laser beam was aligned with the center of the device by determining the location where the intensity of the received signal was maximum. Furthermore, rotational stage rotated the device with respect to the beam axis and provide different incidence angles, 0° ≤ *θ* ≤ 60° for various measurements (Fig. 5(a–d)). In each step of the measurement (i.e., when the polarization or the angle of incidence of the incident beam was changed), the power meter was used to measure the power incident on the device. This was done primarily for complete data collection and for SNR calculation. However, since the polarization sensing capability of the device is not a function of the power density of the incident light, any changes in the irradiance of the illuminating IR light did not impact the measured response of the device.

## Electronic supplementary material

Supplementary Information
