# Supplementary material for: A Wide Dynamic Range Polarization Sensing Long Wave Infrared Detector
Source: Sci Rep. 2017 Dec 12;7:17475. doi: 10.1038/s41598-017-17675-6 (PMC5727214; doi:10.1038/s41598-017-17675-6)
Supplement: Supplementary file 1 — Supplementary Information [file 41598_2017_17675_MOESM1_ESM.pdf]

A Wide Dynamic Range Polarization Sensing Long Wave Infrared Detector

Elham Mohammadi [1], Nader Behdad [1]
[1] University of Wisconsin-Madison, Department of Electrical and Computer Engineering, Madison, WI, 53706, USA
[*] Corresponding author Email: behdad@wisc.edu

Supplementary Information

This file contains 3 supplementary figure.
Captions and legends are also included.

Effect of dielectric thickness:

Supplementary Figure S1. Normalized absorbed power for different dielectric thicknesses. The figure shows the absorbed power at 28.3 THz versus the dielectric thickness. As can be seen, the absorbed power for a thickness of 700 nm is reasonably close to the peak value that occurs for thickness values of 800 nm and 1.4 um.

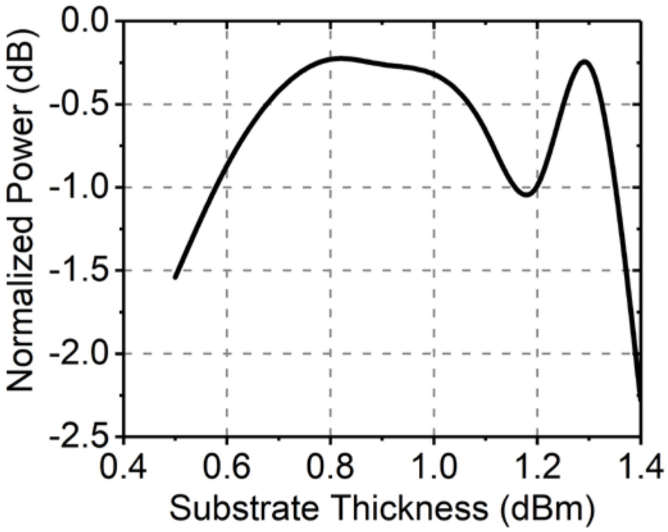

Measurement Setup:

Supplementary Figure S2. Measurement setup. The measurement setup consists of an infrared CO_2 laser, wire grid polarizer to provide desire polarization, beam splitter, power meter, mechanical chopper, DC bias voltage in series with a current preamplifier, and a lock-in amplifier. In each step of the measurement, the power of the incident laser beam is measured using the power meter.

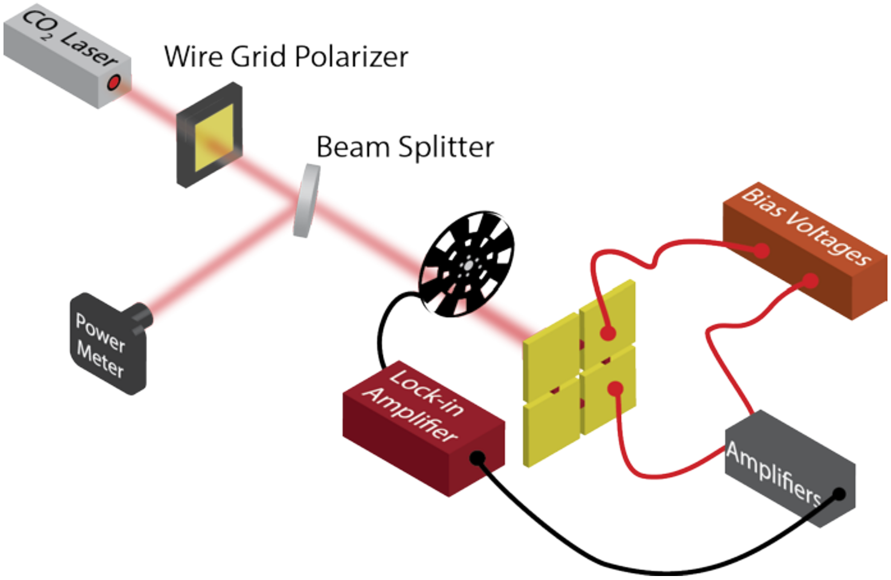

Measurement procedure:

Supplementary Figure S3. Measurement procedure. The resistance combination of bolometers between bias pads. The resistance value of each bolometer is the combination of a constant part (R) and a varying part (ΔR). The constant part is the resistance at room temperature and the varying part is a function of absorbed power. In this figure, we assumed that the bolometers of a single antenna have similar ΔR values.

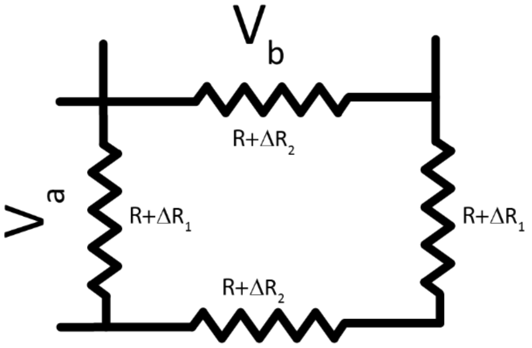

When the bias voltage of V_a is applied, the resistance seen between these two points is:

$$\frac{(R+\Delta R_1)(3R+\Delta R_1+2\Delta R_2)}{4R+2\Delta R_1+2\Delta R_2}$$

Therefore, the measured resistance variation is:

$$\Delta R_a = \frac{(R+\Delta R_1)(3R+\Delta R_1+2\Delta R_2)}{4R+2\Delta R_1+2\Delta R_2} - \frac{3R}{4} \cong \frac{3R^2+4R\times\Delta R_1+2R\times\Delta R_2}{4R+2\Delta R_1+2\Delta R_2} - \frac{3R}{4}$$

$$= \frac{6R^2+8R\times\Delta R_1+4R\times\Delta R_2-6R^2-3R\times\Delta R_1-3R\times\Delta R_2}{2\times(4R+2\Delta R_1+2\Delta R_2)}$$

$$= (\Delta R_1+\frac{\Delta R_2}{5})\times\frac{5R}{2\times(4R+2\Delta R_1+2\Delta R_2)}$$

Using the same equations for V_b,

$$\Delta R_b = (\Delta R_2+\frac{\Delta R_1}{5})\times\frac{5R}{2\times(4R+2\Delta R_1+2\Delta R_2)}$$

Therefore,

$$\Delta R_1 \propto \Delta R_a - \frac{\Delta R_b}{5}, \qquad \Delta R_2 \propto \Delta R_b - \frac{\Delta R_a}{5}$$

$$\frac{\Delta R_1}{\Delta R_2} = \frac{\Delta R_a - \frac{\Delta R_b}{5}}{\Delta R_b - \frac{\Delta R_a}{5}}$$

Noise:

Different noise sources contribute to the measured noise. These noise sources can be classified into three main categories:

1- **Johnson noise:**
Johnson noise is due to the thermal motion of charge carriers in a resistive element. The RMS voltage of Johnson noise is defined: $v_J = \sqrt{4kTR_D\Delta f}$, where k is Boltzmann's constant, T is the temperature in Kelvin, R_D is the resistor value, and Δf is the electrical bandwidth of the measurement. In our measurements, the Johnson noise is due to the resistance of the bolometers and the detector under test and also the noise of internal resistor of current preamplifier.

2- **Shot noise:**
Shot noise is the time domain current fluctuations due to the discreteness of the electron charges. Shot noise is known to occur in solid state devices. However, there are some theories that show their existence in metallic resistance. The RMS of shot noise can be expressed by: $i_s = \sqrt{2qI_{dc}\Delta f}$, where q is the charge of an electron and I_{dc} is dc current. The shot noise is more dominant in active devices such as transistors and in diodes and has lower effect in bolometers.

3- **Flicker noise:**
Flicker noise or $1/f$ noise occurs almost in all electronic devices and can be expressed as $i_f \propto \sqrt{\frac{i_{dc}^2 \Delta f}{f}}$, where f is the frequency in Hz.

By considering $i_{dc} = \frac{V_{dc}}{R_D}$, these three types of noise can be formulized in a general format at the output terminal (This formula is also included in Ref. 1 of supplementary materials.)

$$V_n = \sqrt{A + \frac{B}{R_D} + \frac{C}{R_D^2}} ,$$

where A is the noise associated with measurement equipment, B is a function of thermal noise and shot noise and C is related mostly to flicker noise. To extract the noise of measurement equipment, we have done three measurements with three resistors with known resistance values (see Table S1).

Supplementary Table S1. Noise measurement for various resistors.

R_D(Ω)	V_n (μV)
3.1	3.56
10	1.789
50	0.917

With these measured values, the value of parameters in the noise formula are as follows:

Supplementary Table S2.Calculated values of the parameters in the noise formula.

A=0.336	B=24.4	C=42.5

Since A is the noise of the measurement setup, it can be removed from the measured noise for SNR calculations. The two other parts of the noise formula are mainly due to the Johnson noise and flicker noise. These parts are dependent on the resistor value. In SNR measurement, we have excluded noise from measurement equipment (A). However, all the other noise sources (flicker, Johnson, and shot noise) are considered in our calculations.

References:

1. Bean, Jeffrey A. *Thermal infrared detection using antenna-coupled metal-oxide-metal diodes*. University of Notre Dame, 2009.
